# Adverse childhood experiences and subsequent experiences of intimate partner violence in adulthood: a gender perspective

**DOI:** 10.1017/S2045796024000775

**Published:** 2024-12-10

**Authors:** Zheng Tian, Nan Zhang, Yimiao Li, Yibo Wu, Lan Wang

**Affiliations:** 1 School of Nursing, Tianjin Medical University, Tianjin, China; 2 School of Public Health, Peking University, Beijing, China

**Keywords:** Chinese, intimate partner violence, network analyse, adverse childhood experience

## Abstract

**Aims:**

Investigate the prevalence of adverse childhood experience (ACE) and intimate partner violence (IPV) using a large representative Chinese sample, explore the association mechanism between ACE and adult exposure to IPV and to examine gender differences.

**Methods:**

A total of 21,154 participants were included in this study. The ACE scale was used to assess participants’ exposure to ACE before the age of 18. Participants were evaluated for IPV experienced after the age of 18 using the IPV Scale. Logistic regression model was used to analyse the association between ACE and the risk of IPV exposure in adulthood. Principal component analysis was used to extract the main patterns of ACEs in the Chinese population. Network analyses were employed to identify the most critical types of ACE and IPV, analyse the association mechanisms between ACEs and IPVs, explore gender differences in this association and compare gender differences in the severity of IPVs experienced in adulthood.

**Results:**

Participants with at least one ACE event faced a 215.5% higher risk of IPV compared to those without ACE experiences. In population-wide and gender-specific networks, The ACE and IPV nodes with the highest expected influence are ‘ACE1 (Verbal abuse + physical abuse pattern)’ and ‘IPV5 (Partner compares me to other people and blatantly accuses me, making me feel embarrassed and unsure of myself)’. Positive correlations were found between ‘ACE1 (Verbal abuse + physical abuse pattern)’–‘IPV3 (Partner does not care about me when I am in bad shape [not feeling well or in a bad mood])’, ‘ACE4 (Violent treatment of mother or stepmother + criminal acts in the family pattern)’–‘IPV1 (Partner has ever directly assaulted or hurt me with the help of an instrument)’ and ‘ACE2 (Exposure to sexual assault pattern)’–‘IPV2 (Partner would have physical or sexual contact with me against my will)’, which were the three edges with the highest edge weight values in the ACE pattern and IPV edges. ‘ACE1 (Verbal abuse + physical abuse pattern)’–‘IPV3 (Partner does not care about me when I am in bad shape [not feeling well or in a bad mood])’, ‘ACE2 (Exposure to sexual assault pattern)’–‘IPV2 (Partner would have physical or sexual contact with me against my will)’, ‘ACE4 (Violent treatment of mother or stepmother + criminal acts in the family pattern)’–‘IPV1 (Partner has ever directly assaulted or hurt me with the help of an instrument)’ in the male network and ‘ACE1 (Verbal abuse + physical abuse pattern)’–‘IPV3 (Partner does not care about me when I am in bad shape [not feeling well or in a bad mood])’, ‘ACE4 (Violent treatment of mother or stepmother + criminal acts in the family pattern)’–‘IPV1 (Partner has ever directly assaulted or hurt me with the help of an instrument)’, ‘ACE3 (Substance abuse + mental illness + violent treatment of mother or stepmother pattern)’–‘IPV1 (Partner has ever directly assaulted or hurt me with the help of an instrument)’ in the female network are the three edges with the highest edge weights among the ACE and IPV edges in their networks, respectively, all displaying positive correlations. The strength of ‘IPV3 (Partner does not care about me when I am in bad shape [not feeling well or in a bad mood])’ was higher in the male network than in the female (male = 0.821, female = 0.755, *p* = 0.002). The edge weight values of ‘ACE3 (Substance abuse + mental illness + violent treatment of mother or stepmother pattern)’–‘IPV1 (Partner has ever directly assaulted or hurt me with the help of an instrument)’ (*P* = 0.043) and ‘ACE4 (Violent treatment of mother or stepmother + criminal acts in the family pattern)’–‘IPV1 (Partner has ever directly assaulted or hurt me with the help of an instrument)’ (*P* = 0.032) are greater for females than males.

**Conclusions:**

The most common type of ACE in the Chinese population is verbal violence combined with physical violence, while the predominant type of IPV is verbal violence. Males experience higher levels of emotional neglect from their partners compared to females. The association between witnessing physical violence in childhood and experiencing physical violence from a partner in adulthood is stronger in females than in males. The homotypic continuum between ACE and IPV is a crucial mechanism in understanding intergenerational domestic violence. Enhance economic and educational levels, promote correct parenting concepts, reduce child abuse, establish accurate perceptions of intimate relationships, eliminate shame about violence and further advance gender equality. These efforts are vital for reducing IPV prevalence and breaking the cycle of violence in victims’ lives.

## Introduction

Intimate partner violence (IPV) is a common form of gender-based violence that includes physical, sexual or psychological harm between partners (Bogat *et al.*, 2023). The global prevalence of IPV is estimated to be as high as 27.9%–45.6% (Berry and Monk, 2020). IPV increases the risk of a range of adverse physical and mental health problems such as asthma, digestive disorders, headaches, chronic pain, depression, anxiety and other mental and physical health problems among victims (Rivara *et al.*, 2019) and cumulatively imposes a lifelong economic burden of $3.6 trillion on adult survivors (Peterson *et al.*, 2018), hindering the achievement of the United Nations Sustainable Development Goals (SDGs) (Goal 5 | Department of Economic and Social Affairs 2024).

Adverse childhood experiences (ACEs), also known as childhood adversities, are traumatic events experienced by children or adolescents under the age of 18, including psychological abuse, physical abuse and sexual abuse (Tzouvara *et al.*, 2023). It is estimated that nearly 1 billion children worldwide are exposed to ACE each year (Hillis *et al.*, 2016). As the evidence base related to relationship violence strengthens, an increasing number of studies have found that there is an association between the experience of violence across the lifespan of a victim of violence (Warren *et al.*, 2023), and that victims of ACE are more likely to be victims of IPV (Syed *et al.*, 2023). Early prevention and intervention for ACE would be an effective way to reduce IPV victimization in adulthood (Responding to Intimate Partner Violence and Sexual Violence Against Women 2013; Overview | Domestic violence and abuse 2014; García-Moreno *et al.*, 2015; Preventive Services Task Force *et al.*, 2018; Feltner *et al.*, 2018; Thulin *et al.*, 2021; Working together to safeguard children 2023; The best start for life 2024; WHO, 2023). However, the association between ACE and study outcomes often varies depending on the type of ACE (Fujiwara, 2022; Lu *et al.*, 2020), and the association between different types of ACE and IPV in adulthood has not been fully explored, making the development of interventions challenging.

Rationally clustering ACEs can help reveal the relationship between ACEs and IPV in adulthood and elucidate the underlying mechanisms for effective intervention in IPV. Studies have shown that ACEs tend to cluster, and victims of ACEs often experience multiple ACE events, with multiple types of ACE events often displaying complex patterns of co-occurrence (Lacey and Minnis, 2020). A study found that 36.1% of ACE victims experienced at least two or more types of ACE (Merrick *et al.*, 2019). In previous studies, researchers have often explored the effect of ACE on study outcomes by exploring the effect of each ACE event separately (Sun *et al.*, 2023). For example, Merrick examined the associations between different types of ACEs and adult mental health outcomes separately without adjusting for other ACE effects and found the strongest associations between parental substance abuse and subjects’ own involvement in risky health behaviours (Merrick *et al.*, 2017). Alcalá analysed the impact of different types of ACE on cancer screening and found that participants who had been physically abused in childhood were less likely to participate in prostate, breast or cervical screening (Alcalá *et al.*, 2018). However, exploring the effect of each ACE event separately ignores the simultaneous presence of other types of ACEs, and the association of the single ACE analysed with the outcome variable may be confounded by the presence of other ACEs (Lacey and Minnis, 2020). In addition, some studies summed the number of ACE events to calculate ACE scores, assuming that each ACE event had the same effect on the study outcome (Wakuta *et al.*, 2023). Watson explored the association between ACEs and depression and anxiety during pregnancy using ACE scores and found that participants who had experienced four or more ACEs were at a higher risk of depression and anxiety during pregnancy compared to those who had not (Watson *et al.*, 2024). Lin used ACE scores to analyse the association between ACEs and chronic disease risk in middle-aged and older Chinese adults and found an association between ACEs and the increased risk of various chronic and multiple diseases (Lin *et al.*, 2021). However, the ACE scoring methodology assumes that each adversity is equally important to the outcome, and since ACEs may influence the study results through different combinations of cumulative effects, ignoring specific patterns of co-occurrence of ACEs can similarly bias the results (Lacey and Minnis, 2020; Wang *et al.*, 2023a). Therefore, exploring the relationship between ACE and IPV based on specific ACE combination patterns is essential to further reveal the association mechanism between ACE and IPV and to develop early prevention strategies for IPV.

Moreover, previous studies have tended to examine the relationship between ACE and IPV as a whole, lacking a fine-grained understanding of the category-specific level relationships between them (Cprek *et al.*, 2021; Masiano *et al.*, 2023). Network analysis provides a new research paradigm for exploring the relationship between multiple categories of ACE and IPV. The network structure consists of nodes representing variables and connecting lines representing statistical relationships between variables (Zhang *et al.*, 2023). Compared with traditional statistical models, network analysis has the following advantages: (1) the ability to clarify fine-grained relationships between variables and provide the best targets for effective interventions; (2) the possible avoidance of spurious correlations due to a large number of variables; (3) the ability to visualize interactions between variables and (4) the ability to calculate metrics for evaluating the relative importance (Yang *et al.*, 2023). Thus, network analysis will help to analyse the complex associations between different types of ACE and different types of IPV in adulthood.

Differences in the severity of ACE exposure to IPV exist across gender populations, and studies have shown that women are at higher risk for abusive emotional abuse from their partners than men (Cundiff *et al.*, 2023), while men are at higher risk for physical abuse, neglect and violence during childhood (Polanco-Roman *et al.*, 2021), which may contribute to differences in the association of ACE with IPV across gender populations. Notably, studies have shown that men have a lifetime IPV victimization rate as high as 33.6% (Smith *et al.*, 2024). However, current screening and interventions based on male IPV victims are far from adequate (Park *et al.*, 2021). Further exploring gender differences in the relationship between ACE and IPV exposure in adulthood for different gender populations will help advance knowledge of the impact of relationship violence on different gender populations and promote gender equality.

The SDGs 2015–2030 emphasize that all forms of violence and gender inequality should be eliminated to achieve the SDGs, especially in low- and middle-income countries (SDG Target 5.2.1) (Halkos and Gkampoura, 2021). China is the world’s most populous middle-income country (National Bureau of Statistics, 2023), and exploring IPV prevention and interventions based on a demographically representative sample of Chinese population to reveal gender differences will help to contribute to the achievement of the global SDGs. Currently, IPV remains an urgent social problem in China. Compared with Western countries, empirical results on IPV are scarce in China, and due to sample accessibility, individuals experiencing IPV in rural China have not been adequately assessed in previous studies (Zhang *et al.*, 2021). Therefore, the present study aimed to investigate the prevalence of ACE and IPV based on a large sample of widely covered and representative Chinese data, to explore the association between ACE and exposure to IPV in adulthood and to explore gender differences, in order to provide a basis for in-depth knowledge of the association between ACE and IPV, to formulate strategies for the early management of IPV and to promote gender equality in the study of relationship violence. This study hypothesis: (1) Exposure to ACE increases victims’ risk of IPV in adulthood, and there is a specific pattern of association between different combinations of ACE and different types of IPV. (2) There are differences in the severity of exposure to a specific ACE or a specific IPV across gender populations, and there may be a gender difference in the association of a specific combination of ACE with a specific IPV.

## Methods

### Data source and participants

All data in this study were extracted from the cross-sectional data of the Psychology and Behavior Investigation of Chinese Residents (PBICR) 2023, which is a multi-centre, large-sample, nationwide cross-sectional study. The study was conducted from 20 June 2023 to 20 August 2023, in 150 cities, 480 rural communities (villages) and 320 urban communities across 23 provinces, 5 autonomous regions, 4 municipalities directly under the central government and 2 special administrative regions of China. PBICR 2023 adhered to the Declaration of Helsinki of the World Medical Association, received ethical approval from Shandong Provincial Hospital (SWYX: 2023-198) and was registered in the China Clinical Trial Registry (ChiCTR) (ChiCTR2300072573) (Psychology and Behavior Investigation of Chinese Residents, 2024). All participants in the PBICR received written informed consent prior to participating in the survey (eMethods in Supplement). In this study, 30,054 participants from the PBICR 2023 cross-sectional survey were selected, and 8,900 participants with missing IPV data were excluded, resulting in the inclusion of 21,154 participants. Detailed demographic information is shown in Table 1.

**Table 1.** Comparison of IPV scores of participants with different characteristics

Variables		Mean/*N*	SD/%	*t*/*r*	*P*
Gender (%)	Male	10,279	48.59	−3.291	0.001
	Female	10,875	51.41		
Age (years)		49.93	13.81	0.062	<0.001
BMI (kg/m^2^)		22.79	3.26	−0.023	0.001
Ethnicity (%)	Han ethnic group	19,440	91.90	9.283	<0.001
	Ethnic group	1,714	8.10		
Residence (%)	Cities and towns	13,921	65.81	0.357	0.721
	Rural	7,233	34.19		
Marriage status (%)	Married	19,427	91.84	6.643	<0.001
	Others	1,727	8.16		
Educational status (%)	Bachelor’s degree or above	7,861	37.16	−2.111	0.035
	Below undergraduate level	13,293	62.84		
Occupational status (%)	Incumbency	10,141	47.94	−0.893	0.372
	Others	11,013	52.06		
Availability of liabilities (%)	Yes	8,104	38.31	−17.707	<0.001
	No	13,050	61.69		
Monthly per capita family income (%)	≥4,000	11,180	50.46	0.366	0.038
	<4,000	10,974	49.54		
Suffering from illness (%)	Yes	8,500	40.18	−11.827	<0.001
	No	12,654	59.82		
Self-assessment of family social status (scores)		4.07	1.37	0.101	<0.001
Length of sleep (hours)		7.21	1.49	0.100	<0.001
Sleep quality (%)	Good	17,113	80.90	18.719	<0.001
	Poor	4,041	19.10		
Drinking (%)	Yes	7,753	63.35	−12.735	<0.001
	No	13,401	36.65		
Smoking (%)	Yes	4,851	22.93	−11.297	<0.001
	No	16,303	77.07		

### Research instruments

#### Intimate partner violence

Participants were assessed for IPV at the hands of a partner after the age of 18 years using the IPV Scale, which was developed by the PBICR program group (Understanding and addressing violence against women 2024). The scale covers four dimensions of acts of physical violence, sexual violence, psychological abuse and controlling behaviours. The scale consisted of five entries, each of which was as follows: (1) my partner had directly assaulted or hurt me with the help of an instrument; (2) my partner would have physical or sexual contact with me against my will; (3) my partner did not care about me when I was in a bad state (physically ill or in a bad mood); (4) my partner would go through my cell phone, decide on the way I dressed and limit my interpersonal interactions; (5) my partner compares me with others, blatantly accuses me and makes me feel embarrassed and unsure of myself. The IPV scale uses a 5-point Likert scale to rate each entry, with a score of 1 indicating never, 2 indicating rarely, 3 indicating sometimes, 4 indicating often and 5 indicating almost always. The Cronbach’s alpha coefficient for the IPV scale in this study was 0.937.

#### Adverse childhood experiences

The ACE scale from the PBICR was utilized to assess participants’ exposure to ACE before the age of 18. The scale covers seven dimensions of psychological abuse, physical abuse, exposure to sexual assault, substance abuse, mental illness, violent treatment of mother or stepmother and criminal behaviour in the family, with a total of 17 entries (Felitti *et al.*, 1998). Each entry in the ACE scale was set up with a dichotomous option, with a score of 1 indicating yes and 0 indicating no. A respondent was defined as being exposed to the ACE corresponding to an entry if he or she answered yes to the entry. Scores on the scale range from 0 to 17. In this study, the Cronbach’s alpha coefficient for the ACE scale was 0.854.

### Statistical analysis

The minimum sample size formula for ACE and IPV prevalence and prevalence surveys reported in previous studies was used to calculate the minimum sample size for ACE and IPV prevalence surveys in each province of China (eMethod in Supplement) (Wang *et al.*, 2023b; Yuan *et al.*, 2023), and Tableau 2019.4 was used to draw prevalence maps. SPSS27.0 software was used for data analysis. All the measurements of this study were tested to obey normal distribution. Measurement information was expressed as mean or standard deviation, and count information was expressed as frequency or constitutive ratio. Comparisons between groups of data were made using the *t*-test for two independent samples. Pearson’s correlation was used to analyse the correlation between two continuous variables. Logistic regression model was used to analyse the association between ACE and the risk of IPV exposure in adulthood. Principal component analysis of exploratory factor analysis was used to extract the main patterns of ACEs in the Chinese population. Principal component analysis is a multivariate statistical technique that simplifies the data structure and converts multiple indicators into a few principal components, thus reflecting the main characteristic information of the original data (Tsuchida *et al*., 2022). Each of the principal components in the results of principal component analysis is often used to respond to the most frequently co-occurring patterns of the analysed data (Ebrahimi *et al*., 2023; Gentilotti *et al*., 2023).

R 4.3.1 software was used for network analysis. In the network graph, nodes represent the observed variables, and lines represent the correlation between two nodes; the stronger the relationship between two nodes, the thicker and darker the lines. Green solid lines are used to indicate positive correlations and red solid lines are used to indicate negative correlations. bootnet and qgraph packages are used to perform network estimation, create and draw network graphs (Yang *et al.*, 2022). The mgm package is used to calculate the expected influence (EI) of the network nodes. EI is the centrality index with the highest stability and interpretability that quantifies the influence of the nodes in an undirected network, and a larger EI indicates a higher importance of the nodes in the network (Foygel and Drton, 2010). The bootnet::estimateNetwork function was used to calculate the association between the Pearson correlation coefficient ACE combination and exposure to IPV in adulthood. The least absolute shrinkage and selection operator regularization and the extended Bayesian information criterion (EBIC) were applied for network estimation, and the EBIC was set to 0.5 according to the recommendation of Foygel, in order to reduce the network noise and redundant information and better capture the true correlation between variables (Lin *et al.*, 2021; Xu *et al.*, 2005). The Fruchterman–Reingold algorithm was used for network layout in which strong correlations were placed in the centre of the network and weak correlations were placed in the periphery of the network (Malhotra and Kempegowda, 2023). In this study, a self-help method based on case-dropping subset is used to assess the robustness of the network structure. The method verifies the stability and reliability of the network results by recalculating the centrality metrics of the nodes in the network while removing cases from the original sample. The maximum proportion of cases that can be removed from the sample is indicated by the CS (correlation stability coefficient) value, and a CS > 0.25 indicates that the constructed network structure is stable and reliable (Guo *et al.*, 2023). In addition, this study used Network Comparison Test for network difference comparison, NetworkComparisonTest package was used for this analysis (van Borkulo *et al.*, 2023), which calculates the distribution of the absolute value of all edge weights between the two networks, and the difference in the strength of each edge in order to compare the differences between ACE and IPV networks in different gender populations (Wang *et al.*, 2023c).

A two-sided test was taken for all tests in this study, and differences were considered statistically significant at *P* < 0.05.

## Results

### Baseline characteristics

Among the 21,154 participants included, the mean score for the total IPV score was 20.58 (SD = 5.89) (eTable 1 in Supplement). There were 10,279 (48.59%) males, 10,875 (51.41%) females, and the mean age was 49.93 years (SD = 13.81). There were 19,440 (91.90%) Han ethnic group, 13,921 (65.81%) lived in cities and towns and 19,427 (91.84%) were married (Table1). A total of 5,071 (24.00%) of the participants in the sample suffered from ACE (eTable 2 in Supplement).

### Prevalence of ACE, IPV and ACE combined with IPV in China

The prevalence of ACE, IPV and ACE combined with IPV in selected provinces in China is reported in eTable 3 in Supplement, and the survey of all provinces is shown in eTable 4 in Supplement. eFigure 1 in Supplement shows the map of prevalence of ACE combined with IPV in selected provinces in China. It was found that Guizhou was the province in China with the highest prevalence of ACE, IPV and also the highest prevalence of ACE combined with IPV.

### Association between ACEs and IPV risk

Binary logistic regression models were used to explore the association between ACE and the risk of IPV exposure in adulthood, and after adjusting for the three models, the logistic regression results showed that ACE increased the risk of IPV exposure in adulthood for victims. Participants who experienced at least one ACE event had a 215.5% increased risk of IPV compared to participants who did not have an ACE (OR = 3.155). See Table 2 for details.

**Table 2.** Binary logistic regression analysis of the association between ACEs and IPV

Model adjustment	*β*	SE	Wald *χ*^2^ value	*P*-value	OR (95%CI)
Model 1	1.308	0.035	1434.807	<0.001	3.710 (3.458, 3.960)
Model 2	1.206	0.035	1161.175	<0.001	3.340 (3.116, 3.580)
Model 3	1.149	0.036	1026.615	<0.001	3.155 (2.941, 3.385)

Model 1 was unadjusted; Model 2 was adjusted for sociodemographic factors including gender, age, BMI, ethnicity, place of residence, marital status, education, occupational status, presence of debt, per capita monthly household income, presence of disease and self-assessed social status of the family and Model 3 was adjusted for behavioural-related factors and sociodemographic factors, including all the variables in Model 2, as well as time spent sleeping, quality of sleep, whether or not they drank alcohol and whether or not they smoked.

### Major patterns of exposure to ACEs in China

This study derived four major ACE patterns in the Chinese population, which were ‘ACE1 (Verbal abuse + physical abuse pattern)’, ‘ACE2 (Exposure to sexual assault pattern)’, ‘ACE3 (Substance abuse + mental illness + violent treatment of mother or stepmother pattern)’, ‘ACE4 (Violent treatment of mother or stepmother + criminal acts in the family pattern)’, and the specific composition of each model is shown in eResults and Table 5 in Supplement.

### Network analysis of ACE patterns and IPVs

#### Network estimation and strength centrality

**Figure 1.**
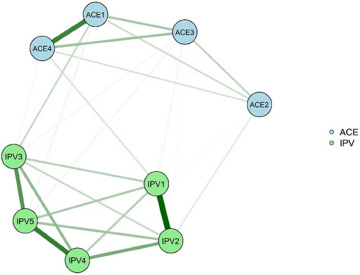
Network of ACE patterns and IPVs.

The network composed of the main patterns of ACEs experienced by the Chinese population and the IPVs suffered in adulthood is shown in Figure 1. The ACE and IPV nodes with the highest EI in the network are ‘ACE1 (Verbal abuse + physical abuse pattern)’ and ‘IPV5 (Partner compares me to other people and blatantly accuses me, making me feel embarrassed and unsure of myself)’ (eFigure 2 in Supplement). There is a positive correlation between ‘ACE1 (Verbal abuse + physical abuse pattern)’–‘IPV3 (Partner does not care about me when I am in bad shape [not feeling well or in a bad mood])’, ‘ACE4 (Violent treatment of mother or stepmother + criminal acts in the family pattern)’–‘IPV1 (Partner has ever directly assaulted or hurt me with the help of an instrument)’ and ‘ACE2 (Exposure to sexual assault pattern)’–‘IPV2 (Partner would have physical or sexual contact with me against my will)’, which are the three edges with the highest edge weight values in the ACE pattern and IPV edges (eTable 6 in Supplement). Network accuracy and stability are shown in eResults, eFigure 3, eFigure 4 and eFigure 5 in Supplement.

### Network analysis of ACE patterns and IPVs in different genders

#### Network estimation and strength centrality of different genders

**Figure 2.**
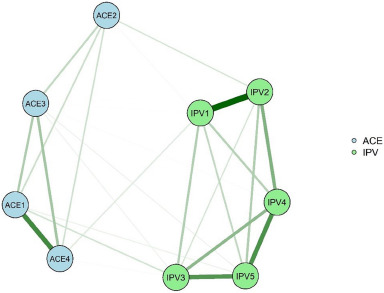
Network of ACE patterns and IPVs in Male.

**Figure 3.**
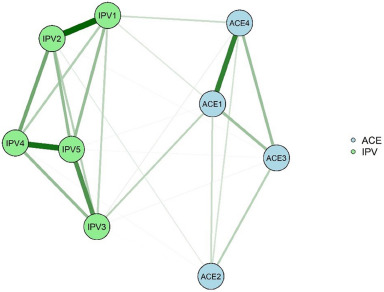
Network of ACE patterns and IPVs in Female.

The main patterns of ACE for males and females and the networks constituted by IPV suffered in adulthood are shown in Figures 2 and 3, respectively. The results of the comparison of the network structure across genders show that there is a significant effect of gender differences on the network (eFigure 6 in Supplement). The ACE and IPV nodes with the highest EI in the male and female networks are ‘ACE1 (Verbal abuse + physical abuse pattern)’ and ‘IPV5 (Partner compares me to other people and blatantly accuses me, making me feel embarrassed and unsure of myself)’ (eFigure 7 and eFigure 8 in Supplement). There is a gender difference in the EI of ‘IPV3 (Partner does not care about me when I am in bad shape [not feeling well or in a bad mood])’ (male = 0.821, female = 0.755, *p* = 0.002) in the male and female networks, with ‘IPV3 (Partner does not care about me when I am in bad shape [not feeling well or in a bad mood])’ being stronger in the male network.

‘ACE1 (Verbal abuse + physical abuse pattern)’–‘IPV3 (Partner does not care about me when I am in bad shape [not feeling well or in a bad mood])’, ‘ACE2 (Exposure to sexual assault pattern)’–‘IPV2 (Partner would have physical or sexual contact with me against my will)’ and ‘ACE4 (Violent treatment of mother or stepmother + criminal acts in the family pattern)’–‘IPV1 (Partner has ever directly assaulted or hurt me with the help of an instrument)’ in the male network and ‘ACE1 (Verbal abuse + physical abuse pattern)’–‘IPV3 (Partner does not care about me when I am in bad shape [not feeling well or in a bad mood])’, ‘ACE4 (Violent treatment of mother or stepmother + criminal acts in the family pattern)’–‘IPV1 (Partner has ever directly assaulted or hurt me with the help of an instrument)’ and ‘ACE3 (Substance abuse + mental illness + violent treatment of mother or stepmother pattern)’–‘IPV1 (Partner has ever directly assaulted or hurt me with the help of an instrument)’ in the female network are the three edges that have the highest weights in the ACE versus IPV edges in their networks, respectively (eTable 7 and eTable 8 in Supplement). The relationship between ACE patterns and IPV suffered in adulthood differed significantly between male and female networks in two edges: ‘ACE3 (Substance abuse + mental illness + violent treatment of mother or stepmother pattern)’–‘IPV1 (Partner has ever directly assaulted or hurt me with the help of an instrument)’ (*P* = 0.043), ‘ACE4 (Violent treatment of mother or stepmother + criminal acts in the family pattern)’–‘IPV1 (Partner has ever directly assaulted or hurt me with the help of an instrument)’ (*P* = 0.032), and greater edge weight values in these two edges for female participants than for male participants. Network accuracy and stability of different genders are shown in eResults, eFigure9, eFigure 10, eFigure 11, eFigure 12, eFigure 13 and eFigure14 in Supplement.

## Discussion

This cross-sectional study based on a whole Chinese population investigated the prevalence of ACE and IPV in China and explored the relationship between ACE and exposure to IPV in adulthood, with the following findings: Guizhou Province in China exhibits the highest prevalence of ACE, IPV and the combined prevalence of ACE and IPV among all provinces. Participants with at least one ACE event faced a 215% higher risk of IPV compared to those without ACE experiences. In population-wide and gender-specific networks, The EI’s highest ACE and IPV nodes are ‘ACE1 (Verbal abuse + physical abuse pattern)’ and ‘IPV5 (Partner compares me to other people and blatantly accuses me, making me feel embarrassed and unsure of myself)’. Positive correlations were found between ‘ACE1 (Verbal abuse + physical abuse pattern)’–‘IPV3 (Partner does not care about me when I am in bad shape [not feeling well or in a bad mood])’, ‘ACE4 (Violent treatment of mother or stepmother + criminal acts in the family pattern)’–‘IPV1 (Partner has ever directly assaulted or hurt me with the help of an instrument)’ and ‘ACE2 (Exposure to sexual assault pattern)’–‘IPV2 (Partner would have physical or sexual contact with me against my will)’, which were the three edges with the highest edge weight values in the ACE pattern and IPV edges. ‘ACE1 (Verbal abuse + physical abuse pattern)’–‘IPV3 (Partner does not care about me when I am in bad shape [not feeling well or in a bad mood])’, ‘ACE2 (Exposure to sexual assault pattern)’–‘IPV2 (Partner would have physical or sexual contact with me against my will)’, ‘ACE4 (Violent treatment of mother or stepmother + criminal acts in the family pattern)’–‘IPV1 (Partner has ever directly assaulted or hurt me with the help of an instrument)’ in the male network and ‘ACE1 (Verbal abuse + physical abuse pattern)’–‘IPV3 (Partner does not care about me when I am in bad shape [not feeling well or in a bad mood])’, ‘ACE4 (Violent treatment of mother or stepmother + criminal acts in the family pattern)’–‘IPV1 (Partner has ever directly assaulted or hurt me with the help of an instrument)’, ‘ACE3 (Substance abuse + mental illness + violent treatment of mother or stepmother pattern)’–‘IPV1 (Partner has ever directly assaulted or hurt me with the help of an instrument)’ in the female network are the three edges with the highest edge weights among the ACE and IPV edges in their networks, respectively, all displaying positive correlations. The strength of ‘IPV3 (Partner does not care about me when I am in bad shape [not feeling well or in a bad mood])’ was higher in the male network than in the female (male = 0.821, female = 0.755, *p* = 0.002). The edge weight values of ‘ACE3 (Substance abuse + mental illness + violent treatment of mother or stepmother pattern)’–‘IPV1 (Partner has ever directly assaulted or hurt me with the help of an instrument)’ (*P* = 0.043) and ‘ACE4 (Violent treatment of mother or stepmother + criminal acts in the family pattern)’–‘IPV1 (Partner has ever directly assaulted or hurt me with the help of an instrument)’ (*P* = 0.032) are greater for females than males.

Low income levels and education are significant risk factors for IPV and ACE (Cao *et al.*, 2023). This study identifies Guizhou as the province with the highest prevalence of ACE and IPV in China, likely due to its low educational attainment and per capita disposable income. The China Statistical Yearbook 2023 reports that Guizhou’s per capita disposable income in 2022 was 25,508.2 yuan, the second lowest among China’s 36 provinces, and the illiterate population was 7.98% of those aged 15 and above, ranking fifth lowest (2023 per capita disposable income released 2024). Previous studies have shown that ACE increases victims’ risk of IPV in adulthood (Syed *et al.*, 2023; Thulin *et al.*, 2021; Zhu *et al.*, 2023), similar to the hypothesis and results of this study. In the network of ACE and IPV for the whole population and different gender populations established separately in this study, it was found that both ‘ACE1 (Verbal abuse + physical abuse pattern)’ and ‘IPV5 (Partner compares me to other people and blatantly accuses me, making me feel embarrassed and unsure of myself)’ were the highest EI ACE and IPV nodes, which indicated that verbal violence combined with physical violence was the most common and important type of ACE and verbal violence was also the most common type of IPV in the Chinese population. This finding is consistent with previous studies (Xu *et al.*, 2005; Zhang *et al.*, 2021; Zhu *et al.*, 2023). The prevalence of ACE and IPV of verbal and physical violence may be related to the educational style of Chinese families. Studies have shown that most Chinese parents educate their children through harsh scolding and physical punishment (Yang and Zhao, 2020; Zhang *et al.*, 2020), and some verbal or physical aggression that can cause children pain is often regarded as ‘discipline’ rather than child abuse. As verbal violence is a form of harm more likely to be ignored violence, its failure to be valued and intervened in may prevent children from developing the ability to recognize and respond to such violence (Liu and Wang, 2018; Sun *et al.*, 2023). Children may view it as the norm with their families and perpetuate verbal violence in their future relationships with their partners (HUO and XIE, 2014; Powers *et al.*, 2020; Sun *et al.*, 2023). Therefore, establishing an accurate public perception of the adverse effects of ACE, moulding correct educational concepts within Chinese families, and advocating healthy and friendly patterns of getting along within the family may be the key to reducing the prevalence of ACE and IPV in China.

Notably, this study identified that ‘ACE1 (Verbal abuse + physical abuse pattern)’–‘IPV3 (Partner does not care about me when I am in bad shape [not feeling well or in a bad mood])’, ‘ACE2 (Exposure to sexual assault pattern)’–‘IPV2 (Partner would have physical or sexual contact with me against my will)’, ‘ACE4 (Violent treatment of mother or stepmother + criminal acts in the family pattern)’–‘IPV1 (Partner has ever directly assaulted or hurt me with the help of an instrument)’ and ‘ACE3 (Substance abuse + mental illness + violent treatment of mother or stepmother pattern)’–‘IPV1 (Partner has ever directly assaulted or hurt me with the help of an instrument)’ had high marginal weight values in the network for the whole population versus the gender-specific population, and that psychological abuse as a child versus psychological abuse as an adult (‘ACE1 [Verbal abuse + physical abuse pattern]’–‘IPV3 [Partner does not care about me when I am in bad shape (not feeling well or in a bad mood)]’), sexual abuse as a child versus sexual abuse as an adult (‘ACE2 [Exposure to sexual assault pattern]’–‘IPV2 [Partner would have physical or sexual contact with me against my will]’), and witnessing a violent beating of a mother or stepmother in childhood versus a violent beating in adulthood (‘ACE4 [Violent treatment of mother or stepmother + criminal acts in the family pattern]’–‘IPV1 [Partner has ever directly assaulted or hurt me with the help of an instrument]’, ‘ACE3 [Substance abuse + mental illness + violent treatment of mother or stepmother pattern]’–‘IPV1 [Partner has ever directly assaulted or hurt me with the help of an instrument]’) had strong positive associations in the network. Previous studies have shown that children who are physically and sexually abused in childhood are more likely to experience physical or sexual violence in adulthood (Miedema *et al.*, 2023; Spencer *et al.*, 2023). These findings, combined with the current study’s results, suggest a potential pattern of associations between ACE endured in childhood and IPV experienced in adulthood along a homotypic continuum (Buffarini *et al.*, 2022; Speranza *et al.*, 2023), meaning that victims may again experience the same forms of violence in adulthood as they did in childhood, this finding is also consistent with the research hypothesis of this study. Attachment theory states that an individual’s experiences in childhood will influence his or her perceptions, attitudes, behaviours and expectations in intimate relationships in adulthood, so that in partnerships, individuals may unconsciously repeat or mimic childhood patterns of relationships with their parents or key caregivers (Knox, 2016; Kochanska *et al.*, 2019; Spencer *et al.*, 2021; Speranza *et al.*, 2021). ACE may bias victims’ perceptions of what they perceive to be intimate relationships in adulthood, making their violence more accepting and misperceive violence as a form of partner care (Knox, 2016). Social learning theory suggests that children learn behaviour by observing and imitating influential role models. When children witness influential role models, such as parents, being violent in the home, they may view violence as an acceptable and effective form of conflict resolution and imitate the same violent behaviours they experience in future intimate relationships (Powers *et al.*, 2020). Thus, the pattern of association between ACE and IPV homotypic continuity may be an important mechanism for intergenerational domestic violence behaviour. Integrating past experiences to enable victims to recognize the mechanisms by which ACE plays a role in IPV in adulthood, developing victims’ ability to reflect on their own and others’ behaviours and psychological states and establishing their correct perceptions of what constitutes a healthy intimate relationship may be the key to freeing victims from the vicious cycle of violence in their lives.

In this study, gender differences in the network structure of ACE and IPV were observed, this finding is also consistent with the research hypothesis of this study. In the male network, the intensity of ‘IPV3 (Partner does not care about me when I am in bad shape [not feeling well or in a bad mood])’ was higher than that in females, indicating that men experience more emotional neglect from their partners compared to women. Previous studies support this observation, showing that men are more likely to endure psychological abuse from their partners than women (Centers for Disease Control and Prevention 2014). Shame Theory states that men tend to demonstrate their resilience and independence as they are socialized, and that men are more likely to avoid expressing their need for support from their partners when faced with situations that may expose their feelings of dependence and vulnerability, which may be an important reason why men are more likely to be emotionally neglected (Xie, 2023). In addition, this study found that the edge weight values of ‘ACE3 (Substance abuse + mental illness + violent treatment of mother or stepmother pattern)’–‘IPV1 (Partner has ever directly assaulted or hurt me with the help of an instrument)’ and ‘ACE4 (Violent treatment of mother or stepmother + criminal acts in the family pattern)’–‘IPV1 (Partner has ever directly assaulted or hurt me with the help of an instrument)’ were greater in the female network compared to males. This implies that in the female population, the association between seeing one’s mother battered in childhood and being battered by one’s partner in adulthood is stronger than in males. Studies have shown that women are at higher risk of physical IPV than men, and that ACE has a greater adverse effect on women than men (Clemens *et al.*, 2023; Polanco-Roman *et al.*, 2021; Thulin *et al.*, 2021). The violence suffered by Chinese women is closely related to the deep-rooted patriarchal values of society (Xu *et al.*, 2005). In societies with deep-rooted patriarchy, men have the supremacy in the family and dominance over their partners, and their aggressive behaviours are often seen as a means of maintaining family status and resolving disputes (Sinha *et al.*, 2023). Children who grow up rationalizing violent behaviour under the influence of patriarchal beliefs are likely to perpetuate the same physical violence against females in future intimate relationships (Fujiwara, 2022). It is worth noting that, due to the traditional Chinese culture of ‘face’, which emphasizes the importance of individual self-esteem and social expectations in interpersonal relationships, most of the physical violence suffered by women is regarded as private and does not receive timely intervention (Chen *et al.*, 2023; Xu *et al.*, 2005). Eliminating the stigma of violence against victims, stopping the violence in a timely manner and educating society about family life, making gender equality and respect for women mainstream values and making children question gender stereotypes may help to eliminate gender-based violence.

There are some limitations to this study. First, all participants in this study were Chinese, which limits the generalizability of the findings. Second, the questionnaire content of this study was all self-reported by the participants, which means that both the exposures and the results of the study may have been affected by misclassification, recall difficulties, recall bias and response style bias. Third, because this was a cross-sectional observational study based on cross-sectional data, it was not possible to construct a directional network, and even with the addition of the age of recollection in the retrospective collection, there may still be some limitations in the causal inference of this study, but the effects of reverse causality and potential confounders could not be ruled out despite adjusting for many confounders. Fourth, the emphasis of traditional Chinese values on harmonious and close family relationships, as well as the stigmatization of victims of violence such as sexual violence in traditional concepts, may have biased participants’ underreporting of ACE versus IPV. Fifth, this research hypothesis is exploratory and does not provide an explicit a priori hypothesis on the relationship between ACE and IPV based on theory. Future studies may explicitly state the expected hypotheses based on reliable theories based on the results of this study to enhance the transparency and scientific validity of the research objectives.

## Conclusion

This study shows that Guizhou is the province with the highest prevalence of ACE and IPV in China, and the most common type of ACE in the Chinese population is verbal violence combined with physical violence, and the most common type of IPV is verbal violence. Males suffered more from partner emotional neglect than females. The association between witnessing physical violence in childhood and experiencing physical violence from a partner in adulthood was stronger in females than in males. The pattern of association between ACE and IPV homotypic continuum may be an important mechanism for intergenerational domestic violence behaviour. Considering the ACE and IPV homotypic continuum association pattern and developing interventions to further reduce IPV prevalence based on areas of high ACE and IPV prevalence is important for victims to escape from the vicious cycle of violence in their lives.

## Supporting information

Tian et al. supplementary material Tian et al. supplementary material

## Data Availability

The datasets generated during the current study are available from the corresponding author with permission of the principal investigators.

## Supplementary material

The supplementary material for this article can be found at https://doi.org/10.1017/S2045796024000775.
