# Supplementary material for: Adverse childhood experiences and subsequent experiences of intimate partner violence in adulthood: a gender perspective
Source: Epidemiol Psychiatr Sci. 2024 Dec 10;33:e75. doi: 10.1017/S2045796024000775 (PMC11669798; doi:10.1017/S2045796024000775)
Supplement: Tian et al. supplementary material [file S2045796024000775sup001.docx]

**Supplementary Content**

**eMethods**

**eResults**

**eTable 1. IPV scores of the participants**

**eTable 2. Comparison of IPV scores of participants with different ACE status**

**eTable 3. Prevalence of ACE, IPV, and ACE combined with IPV in selected provinces of China**

**eTable 4. Prevalence of ACE, IPV, and ACE combined with IPV in 36 provinces in China**

**eTable 5. Major ACE patterns in Chinese population**

**eTable 6. Weighted adjacency matrix of the ACE patterns and IPVs**

**eTable 7. Weighted adjacency matrix of ACE patterns and IPVs in Male**

**eTable 8. Weighted adjacency matrix of ACE patterns and IPVs in Female**

**eFigure 1. Prevalence of ACE combined with IPV in selected provinces of China**

**eFigure 2. EI values of nodes in network**

**eFigure 3. Estimating the stability of the network structure using the case-drop subset bootstrap method**

**eFigure 4. Difference-in-difference test for node EI**

**eFigure 5. Bootstrap Difference Test of Network Edge Weights**

**eFigure 6. Comparison of network attributes of participants in different gender**

**eFigure 7. EI values of nodes in Male network**

**eFigure 8. EI values of nodes in Female network**

**eFigure 9. Estimating the stability of the network structure using the case-drop subset bootstrap method in Male**

**eFigure 10. Estimating the stability of the network structure using the case-drop subset bootstrap method in Female**

**eFigure 11. Difference-in-difference test for node EI in Male**

**eFigure 12. Difference-in-difference test for node EI in Female**

**eFigure 13. Bootstrap Difference Test of Network Edge Weights in Male**

**eFigure 14. Bootstrap Difference Test of Network Edge Weights in Female**

**eReferences**

**eMethods**

**Data Source and Participants**

PBICR jointly sponsored by the Department of Social Medicine and Health Education, School of Public Health, Peking University; the Institute of Health Yangtze River Delta Research, Shanghai Jiaotong University; and the Research Institute and Shandong Provincial Hospital. During the questionnaire design stage, PBICR collaborated with authoritative experts in related fields, conducted more than 30 expert consultations, and carried out three rounds of pre-surveys before the official survey. PBICR investigators underwent strict screening and auditing processes and participated in five rounds of online training before the official survey to ensure the quality of the collected questionnaires.

The inclusion and exclusion criteria for participants were as follows. Inclusion criteria: ①Age ≥18 years old; ②Nationality of the People's Republic of China; ③Resident population of China (annual time away from home ≤1 month); ④Able to complete the online questionnaire independently or with the help of the investigator; ⑤Able to understand the meaning of each entry in the questionnaire. Exclusion Criteria: ①Individuals who are delirious or mentally abnormal; ②Individuals with cognitive impairment; ③Individuals participating in other similar research projects or who have participated in PBICR surveys in previous years; ④Individuals who do not wish to participate in this study. (Note: Information about mental illnesses such as cognitive dysfunction of the respondents was obtained from records in the system of community health centers and self-reports by the respondents).

**Assessment of Covariates**

In this study, gender, age, BMI, ethnicity, place of residence, marital status, education, occupational status, presence of debt, per capita monthly household income, presence of disease, self-rated social status of the family, sleep duration, quality of sleep, presence of alcohol consumption, and presence of smoking. These were used as covariates in the data analysis. Married" in marital status includes first marriage with spouse, remarriage with spouse, remarriage with spouse, and "other" includes divorced, widowed, and unmarried. In the education level, bachelor's degree, master's degree, and doctoral degree are defined as bachelor's degree and above, while junior college, high school, middle school, junior high school, elementary school, and no formal education are defined as below bachelor's degree. Other" in occupational status includes student, retired/retired, no regular occupation (or freelance), unemployed/unemployed, and unemployed. Self-rated family social status was a visual analog scale ranging from 1-7, with higher scores indicating higher self-rated family social status. In sleep quality, self-reported overall sleep was characterized as "good" sleep quality if it was very good or better, and "poor" sleep quality if it was very poor or worse. For drinking status, past drinking, current drinking, and always drinking were defined as "drinking," and never drinking was defined as "not drinking. For smoking status, ever smoked, smoked regular cigarettes, smoked e-cigarettes, smoked e-cigarettes and regular cigarettes were defined as "smoking", and never smoked was defined as "not smoking".

**Statistical analysis**

Studies have shown that the prevalence of ACE in the Chinese population is 80.9% and the prevalence of IPV is 43%.(Wang *et al*., 2023; Yuan *et al*., 2023) Using the sample size calculation formula: n$=\frac{Z^{2}\times P\times(1-P)}{E^{2}}$ The minimum sample sizes for ACE and IPV prevalence surveys in each province in China were calculated, where Z = 1.96 and E = 0.05. The minimum sample sizes required for ACE prevalence surveys in the Chinese region were calculated to be 238, and for IPV prevalence surveys to be 377.

**eResults**

**Major patterns of exposure to ACEs in China**

The result of the applicability test of exploratory factor analysis, KMO = 0.887, indicated that the ACEs were suitable for principal component analysis. The result of the Bartlett's test of sphericity showed χ2 = 135526.289 (p < 0.001), which indicated a strong correlation between the ACEs. Based on Kaiser's criteria, this study decided to retain four factors with a cumulative contribution of 60.78%.

**Network accuracy and stability**

The stability test for network analysis shows CS=0.75. this means that the network structure does not change significantly even if 75% of the samples are discarded (eFigure 3 in Supplement). The correlation between the mean value of the EI of the drawn subsamples and the centrality index of the original samples tends to decrease slowly with the reduction of the drawn sample size, which predicts a better stability of the EI. bootstrap the difference test shows that the difference test of most node EIs is statistically significant (eFigure 4 in Supplement) In addition, this study proved the accurate estimation of edge weights by narrow bootstrap 95% confidence intervals, and the bootstrap difference test for edge weights are shown in (eFigure 5 in Supplement).

**Network estimation and strength centrality of different genders**

The networks composed of the main patterns of ACE and IPV suffered in adulthood for males and females are shown in Figure 2 and Figure 3, respectively. The results of the comparison of the network structure across genders show that there is a significant effect of gender differences on the network, the distribution of edge weights (M = 0.095; P = 0.002) and overall strength (S = 0.082; P = 0.005) changed significantly between network models when comparisons were made (eFigure 6 in Supplement).

**Network accuracy and stability of different genders**

The stability test for the male network analysis shows CS=0.75 and the stability test for the female network shows CS=0.75, indicating that the centrality indexes are more stable for males and females (eFigure9 and eFigure10 in Supplement). the bootstrap difference test showed that most of the difference tests of node EI were statistically significant (eFigure 11 and eFigure 12 in Supplement). In addition, this study proved the accurate estimation of edge weights by narrow bootstrap 95% confidence intervals (eFigure 13 and eFigure 14 in Supplement).

**eTable**

**eTable 1. IPV scores of the participants**

| Variables |  | Mean | SD |
| --- | --- | --- | --- |
| Types of IPV suffered (scores) | Total | 20.58 | 5.89 |
|  | Partner has ever directly assaulted or hurt me with the help of an instrument | 4.31 | 1.37 |
|  | Partner would have physical or sexual contact with me against my will | 4.30 | 1.38 |
|  | Partner does not care about me when I am in bad shape (not feeling well or in a bad mood) | 3.78 | 1.64 |
|  | Partner will go through my cell phone, decide how I dress and limit my social interactions | 4.12 | 1.51 |
|  | Partner compares me to other people and blatantly accuses me, making me feel embarrassed and unsure of myself | 4.06 | 1.53 |

**eTable 2. Comparison of IPV scores of participants with different ACE status**

| Variables |  | Mean/N | SD/% | t/r | P |
| --- | --- | --- | --- | --- | --- |
| Has suffered from ACE | Yes | 5071 | 24.00 | -37.788 | ＜0.001 |
|  | No | 16083 | 76.00 |  |  |
| Cursing, insulting or belittling you(%) | Yes | 2730 | 12.91 | -29.480 | ＜0.001 |
|  | No | 18424 | 87.09 |  |  |
| Engaging in behavior that puts you in fear of physical harm (%) | Yes | 1651 | 7.80 | -25.769 | ＜0.001 |
|  | No | 19503 | 92.20 |  |  |
| Pushing, grabbing, squeezing or slapping you (%) | Yes | 1807 | 8.54 | -22.954 | ＜0.001 |
|  | No | 19347 | 91.46 |  |  |
| Hitting you hard enough to leave marks or bruises (%) | Yes | 1237 | 94.15 | -21.124 | ＜0.001 |
|  | No | 19917 | 5.85 |  |  |
| Touching or fondling you in a sexual way (%) | Yes | 708 | 3.35 | -17.445 | ＜0.001 |
|  | No | 20446 | 96.65 |  |  |
| Touching their body in a sexual way (%) | Yes | 454 | 2.15 | -15.146 | ＜0.001 |
|  | No | 20700 | 97.85 |  |  |
| Trying to have oral, anal or vaginal sex with you (%) | Yes | 387 | 1.83 | -14.781 | ＜0.001 |
|  | No | 20767 | 98.17 |  |  |
| Actually having oral, anal or vaginal sex with you (%) | Yes | 324 | 1.53 | -14.323 | ＜0.001 |
|  | No | 20830 | 98.47 |  |  |
| Living with someone who has a drinking problem (%) | Yes | 1351 | 6.39 | -21.940 | ＜0.001 |
|  | No | 19803 | 93.61 |  |  |
| Living with someone who uses drugs (%) | Yes | 192 | 0.91 | -12.439 | ＜0.001 |
|  | No | 20962 | 99.09 |  |  |
| Family member suffers from depression or mental illness (%) | Yes | 20518 | 96.99 | -16.315 | ＜0.001 |
|  | No | 636 | 3.01 |  |  |
| Family member attempted suicide (%) | Yes | 20733 | 98.01 | -16.693 | ＜0.001 |
|  | No | 421 | 1.99 |  |  |
| A family member has been in jail (%) | Yes | 417 | 1.97 | -12.350 | ＜0.001 |
|  | No | 20737 | 98.03 |  |  |
| Mother or stepmother pushed, grabbed, slapped, or stoned (%) | Yes | 1409 | 6.66 | -21.906 | ＜0.001 |
|  | No | 19745 | 93.34 |  |  |
| Mother or stepmother kicked, bitten, or hit with fists, hard objects (%) | Yes | 909 | 4.30 | -22.256 | ＜0.001 |
|  | No | 20245 | 95.70 |  |  |
| Mother or stepmother was repeatedly hit within a few minutes of each other (%) | Yes | 752 | 3.55 | -20.914 | ＜0.001 |
|  | No | 20402 | 96.45 |  |  |
| Mother or stepmother has been threatened or hurt with a knife (%) | Yes | 309 | 98.54 | -15.054 | ＜0.001 |
|  | No | 20845 | 1.46 |  |  |

**eTable 3. Prevalence of ACE, IPV, and ACE combined with IPV in selected provinces of China**

| Province | Number of Total investigators | Number of ACE investigators | Number of IPV investigators | Number of ACE combined IPV investigations | prevalence of ACE | prevalence of IPV | Combined prevalence of ACE and IPV |
| --- | --- | --- | --- | --- | --- | --- | --- |
| Jilin | 387 | 56 | 140 | 38 | 14.47% | 36.18% | 9.82% |
| Fujian | 472 | 94 | 186 | 52 | 19.92% | 39.41% | 11.02% |
| Zhejiang | 587 | 100 | 241 | 67 | 17.04% | 41.06% | 11.41% |
| Shandong | 1041 | 191 | 432 | 121 | 18.35% | 41.50% | 11.62% |
| Henan | 930 | 162 | 362 | 115 | 17.42% | 38.92% | 12.37% |
| Jiangsu | 1300 | 277 | 511 | 176 | 21.31% | 39.31% | 13.54% |
| Inner Mongolia | 909 | 185 | 377 | 125 | 20.35% | 41.47% | 13.75% |
| Anhui | 1200 | 240 | 566 | 167 | 20.00% | 47.17% | 13.92% |
| Hunan | 584 | 136 | 290 | 94 | 23.29% | 49.66% | 16.10% |
| Liaoning | 557 | 127 | 226 | 90 | 22.80% | 40.57% | 16.16% |
| Chongqing | 586 | 172 | 244 | 96 | 29.35% | 41.64% | 16.38% |
| Hebei | 436 | 98 | 211 | 73 | 22.48% | 48.39% | 16.74% |
| Sichuan | 3735 | 954 | 1597 | 635 | 25.54% | 42.76% | 17.00% |
| Yunnan | 557 | 146 | 263 | 100 | 26.21% | 47.22% | 17.95% |
| Guangxi | 753 | 185 | 367 | 136 | 24.57% | 48.74% | 18.06% |
| Guangdong | 1662 | 434 | 752 | 318 | 26.11% | 45.25% | 19.13% |
| Shaanxi | 418 | 109 | 217 | 81 | 26.08% | 51.91% | 19.38% |
| Jiangxi | 1538 | 397 | 834 | 300 | 25.81% | 54.23% | 19.51% |
| Hainan | 969 | 310 | 481 | 231 | 31.99% | 49.64% | 23.84% |
| Guizhou | 491 | 162 | 286 | 121 | 32.99% | 58.25% | 24.64% |

This table reports the provinces that meet the minimum sample size requirements for ACE and IPV prevalence surveys, which are calculated in the Statistical analysis section of eMethods on.

**eTable 4. Prevalence of ACE, IPV, and ACE combined with IPV in 36 provinces in China**

| Province | Number of Total investigators | Number of ACE investigators | Number of IPV investigators | Number of ACE combined IPV investigations | prevalence of ACE | prevalence of IPV | Combined prevalence of ACE and IPV |
| --- | --- | --- | --- | --- | --- | --- | --- |
| Sichuan | 3735 | 954 | 1597 | 635 | 25.54% | 42.76% | 17.00% |
| Guangdong | 1662 | 434 | 752 | 318 | 26.11% | 45.25% | 19.13% |
| Jiangxi | 1538 | 397 | 834 | 300 | 25.81% | 54.23% | 19.51% |
| Jiangsu | 1300 | 277 | 511 | 176 | 21.31% | 39.31% | 13.54% |
| Anhui | 1200 | 240 | 566 | 167 | 20.00% | 47.17% | 13.92% |
| Shandong | 1041 | 191 | 432 | 121 | 18.35% | 41.50% | 11.62% |
| Hainan | 969 | 310 | 481 | 231 | 31.99% | 49.64% | 23.84% |
| Henan | 930 | 162 | 362 | 115 | 17.42% | 38.92% | 12.37% |
| Inner Mongolia | 909 | 185 | 377 | 125 | 20.35% | 41.47% | 13.75% |
| Guangxi | 753 | 185 | 367 | 136 | 24.57% | 48.74% | 18.06% |
| Zhejiang | 587 | 100 | 241 | 67 | 17.04% | 41.06% | 11.41% |
| Chongqing | 586 | 172 | 244 | 96 | 29.35% | 41.64% | 16.38% |
| Hunan | 584 | 136 | 290 | 94 | 23.29% | 49.66% | 16.10% |
| Liaoning | 557 | 127 | 226 | 90 | 22.80% | 40.57% | 16.16% |
| Yunnan | 557 | 146 | 263 | 100 | 26.21% | 47.22% | 17.95% |
| Guizhou | 491 | 162 | 286 | 121 | 32.99% | 58.25% | 24.64% |
| Fujian | 472 | 94 | 186 | 52 | 19.92% | 39.41% | 11.02% |
| Hebei | 436 | 98 | 211 | 73 | 22.48% | 48.39% | 16.74% |
| Shaanxi | 418 | 109 | 217 | 81 | 26.08% | 51.91% | 19.38% |
| Jilin | 387 | 56 | 140 | 38 | 14.47% | 36.18% | 9.82% |
| Gansu | 339 | 107 | 200 | 89 | 31.56% | 59.00% | 26.25% |
| Shanxi | 279 | 72 | 148 | 49 | 25.81% | 53.05% | 17.56% |
| Beijing | 231 | 64 | 123 | 47 | 27.71% | 53.25% | 20.35% |
| Xinjiang | 198 | 40 | 75 | 26 | 20.20% | 37.88% | 13.13% |
| Ningxia | 179 | 38 | 91 | 26 | 21.23% | 50.84% | 14.53% |
| Hubei | 168 | 44 | 70 | 34 | 26.19% | 41.67% | 20.24% |
| Tianjin | 143 | 31 | 63 | 21 | 21.68% | 44.06% | 14.69% |
| Shanghai | 135 | 29 | 84 | 22 | 21.48% | 62.22% | 16.30% |
| Qinghai | 111 | 26 | 66 | 20 | 23.42% | 59.46% | 18.02% |
| Macao | 96 | 40 | 58 | 26 | 41.67% | 60.42% | 27.08% |
| Heilongjiang | 91 | 18 | 42 | 9 | 19.78% | 46.15% | 9.89% |
| Tibet | 49 | 20 | 30 | 14 | 40.82% | 61.22% | 28.57% |
| Hong Kong | 19 | 5 | 8 | 2 | 26.32% | 42.11% | 10.53% |
| Taiwan | 4 | 0 | 4 | 0 | 0.00% | 100.00% | 0.00% |

This table reports the prevalence of ACE and IPV in all Chinese provinces included in the PBICR 2023 database.

**eTable 5. Major ACE patterns in Chinese population**

| **ID** | **ACE pattern** | **ACE category** | **Factor load** |
| --- | --- | --- | --- |
| ACE1 | Verbal abuse + physical abuse | Cursing, insulting or belittling you | 0.783 |
|  |  | Engaging in behavior that puts you in fear of physical harm | 0.766 |
|  |  | Pushing, grabbing, squeezing or slapping you | 0.757 |
|  |  | Hitting you hard enough to leave marks or bruises | 0.709 |
|  |  |  |  |
| ACE2 | Exposure to sexual assault | Touching or fondling you in a sexual way | 0.850 |
|  |  | Touching their body in a sexual way | 0.847 |
|  |  | Trying to have oral, anal or vaginal sex with you | 0.821 |
|  |  | Actually having oral, anal or vaginal sex with you | 0.769 |
|  |  |  |  |
| ACE3 | Substance abuse + mental illness + violent treatment of mother or stepmother | Living with someone who has a drinking problem | 0.733 |
|  |  | Living with someone who uses drugs | 0.725 |
|  |  | Family member suffers from depression or mental illness | 0.617 |
|  |  | Family member attempted suicide | 0.591 |
|  |  | Mother or stepmother pushed, grabbed, slapped, or stoned | 0.435 |
| ACE4 | Violent treatment of mother or stepmother + criminal acts in the family |  |  |
|  |  | Mother or stepmother kicked, bitten, or hit with fists, hard objects | 0.748 |
|  |  | Mother or stepmother was repeatedly hit within a few minutes of each other | 0.728 |
|  |  | Mother or stepmother has been threatened or hurt with a knife | 0.658 |
|  |  | A family member has been in jail | 0.592 |

**eTable 6. Weighted adjacency matrix of the ACE patterns and IPVs**

|  | IPV1 | IPV2 | IPV3 | IPV4 | IPV5 | ACE1 | ACE2 | ACE3 | ACE4 |
| --- | --- | --- | --- | --- | --- | --- | --- | --- | --- |
| IPV1 | 0.000 | 0.403 | 0.117 | 0.125 | 0.134 | 0.002 | 0.003 | 0.026 | 0.063 |
| IPV2 | 0.403 | 0.000 | 0.096 | 0.215 | 0.150 | 0.000 | 0.052 | 0.000 | -0.002 |
| IPV3 | 0.117 | 0.096 | 0.000 | 0.177 | 0.276 | 0.090 | 0.003 | 0.023 | 0.018 |
| IPV4 | 0.125 | 0.215 | 0.177 | 0.000 | 0.331 | 0.001 | 0.014 | 0.003 | -0.003 |
| IPV5 | 0.134 | 0.150 | 0.276 | 0.331 | 0.000 | 0.027 | 0.001 | 0.026 | 0.003 |
| ACE1 | 0.002 | 0.000 | 0.090 | 0.001 | 0.027 | 0.000 | 0.084 | 0.144 | 0.315 |
| ACE2 | 0.003 | 0.052 | 0.003 | 0.014 | 0.001 | 0.084 | 0.000 | 0.104 | 0.074 |
| ACE3 | 0.026 | 0.000 | 0.023 | 0.003 | 0.026 | 0.144 | 0.104 | 0.000 | 0.154 |
| ACE4 | 0.063 | -0.002 | 0.018 | -0.003 | 0.003 | 0.315 | 0.074 | 0.154 | 0.000 |

**eTable 7. Weighted adjacency matrix of ACE patterns and IPVs in Male**

|  | IPV1 | IPV2 | IPV3 | IPV4 | IPV5 | ACE1 | ACE2 | ACE3 | ACE4 |
| --- | --- | --- | --- | --- | --- | --- | --- | --- | --- |
| IPV1 | 0.000 | 0.439 | 0.137 | 0.131 | 0.110 | -0.002 | 0.003 | 0.005 | 0.046 |
| IPV2 | 0.439 | 0.000 | 0.074 | 0.220 | 0.140 | -0.002 | 0.064 | 0.005 | -0.001 |
| IPV3 | 0.137 | 0.074 | 0.000 | 0.201 | 0.298 | 0.077 | 0.000 | 0.029 | 0.010 |
| IPV4 | 0.131 | 0.220 | 0.201 | 0.000 | 0.311 | 0.002 | 0.013 | 0.008 | 0.000 |
| IPV5 | 0.110 | 0.140 | 0.298 | 0.311 | 0.000 | 0.034 | 0.004 | 0.031 | 0.002 |
| ACE1 | -0.002 | -0.002 | 0.077 | 0.002 | 0.034 | 0.000 | 0.088 | 0.143 | 0.329 |
| ACE2 | 0.003 | 0.064 | 0.000 | 0.013 | 0.004 | 0.088 | 0.000 | 0.103 | 0.082 |
| ACE3 | 0.005 | 0.005 | 0.029 | 0.008 | 0.031 | 0.143 | 0.103 | 0.000 | 0.158 |
| ACE4 | 0.046 | -0.001 | 0.010 | 0.000 | 0.002 | 0.329 | 0.082 | 0.158 | 0.000 |

**eTable 8. Weighted adjacency matrix of ACE patterns and IPVs in Female**

|  | IPV1 | IPV2 | IPV3 | IPV4 | IPV5 | ACE1 | ACE2 | ACE3 | ACE4 |
| --- | --- | --- | --- | --- | --- | --- | --- | --- | --- |
| IPV1 | 0.000 | 0.367 | 0.099 | 0.115 | 0.155 | 0.011 | 0.004 | 0.049 | 0.076 |
| IPV2 | 0.367 | 0.000 | 0.112 | 0.211 | 0.164 | 0.002 | 0.038 | 0.014 | -0.002 |
| IPV3 | 0.099 | 0.112 | 0.000 | 0.159 | 0.259 | 0.099 | 0.007 | 0.017 | 0.028 |
| IPV4 | 0.115 | 0.211 | 0.159 | 0.000 | 0.341 | 0.002 | 0.013 | 0.000 | -0.006 |
| IPV5 | 0.155 | 0.164 | 0.259 | 0.341 | 0.000 | 0.163 | 0.000 | 0.017 | 0.005 |
| ACE1 | 0.011 | 0.002 | 0.099 | 0.002 | 0.163 | 0.000 | 0.080 | 0.142 | 0.298 |
| ACE2 | 0.004 | 0.038 | 0.007 | 0.013 | 0.000 | 0.080 | 0.000 | 0.103 | 0.065 |
| ACE3 | 0.049 | 0.014 | 0.017 | 0.000 | 0.017 | 0.142 | 0.103 | 0.000 | 0.148 |
| ACE4 | 0.076 | -0.002 | 0.028 | -0.006 | 0.005 | 0.298 | 0.065 | 0.148 | 0.000 |

**eFigure**

**
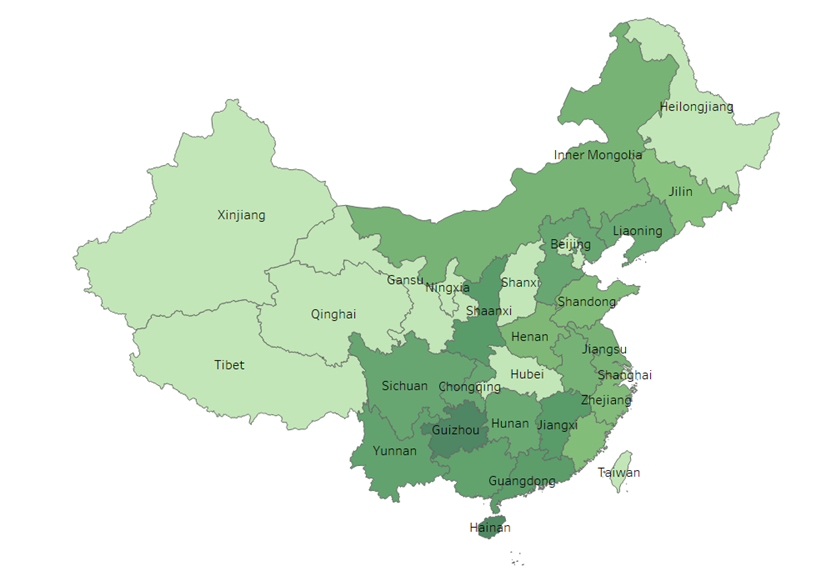
**

**eFigure 1. Prevalence of ACE combined with IPV in selected provinces of China**

**
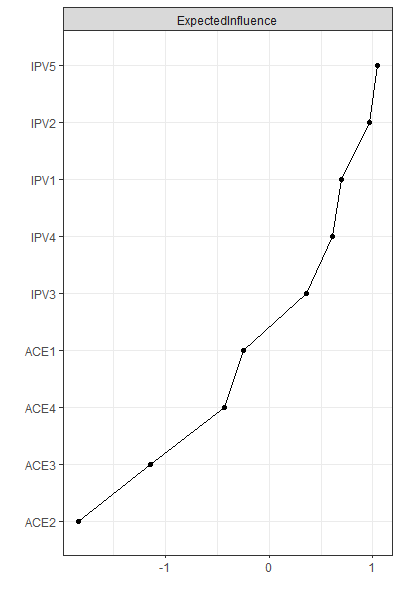
**

**eFigure 2. EI values of nodes in network**

**
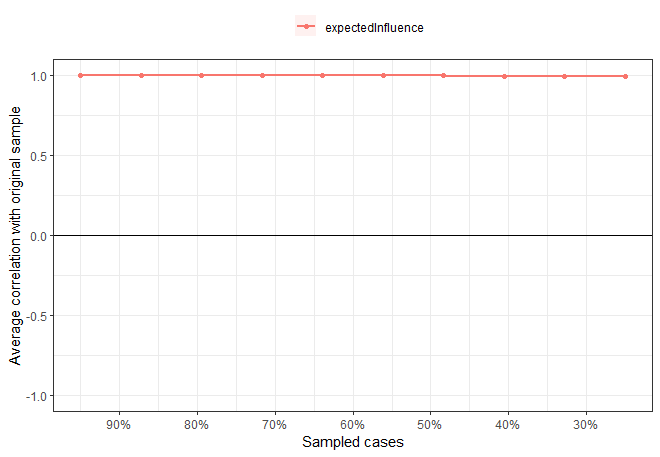
**

**eFigure 3. Estimating the stability of the network structure using the case-drop subset bootstrap method**

**
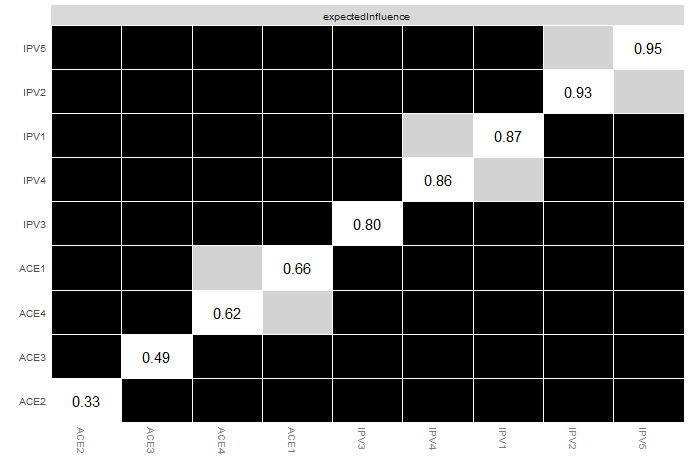
**

**eFigure 4. Difference-in-difference test for node EI**

Each square in the graph represents the difference between nodes, black represents the presence of differences between nodes, gray represents the absence of differences, and the values in the white squares represent the specific values of the corresponding intensities of the nodes


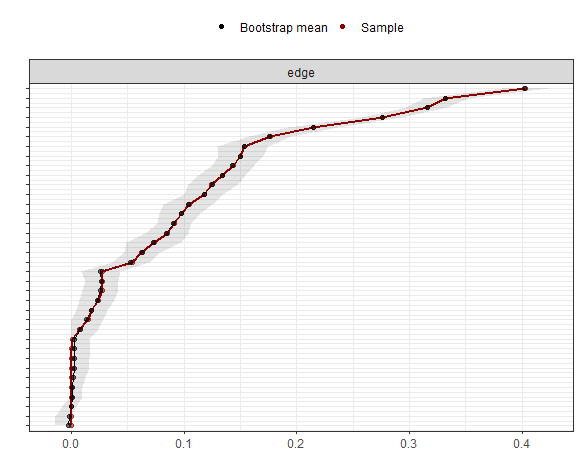


**eFigure 5. Bootstrap Difference Test of Network Edge Weights**


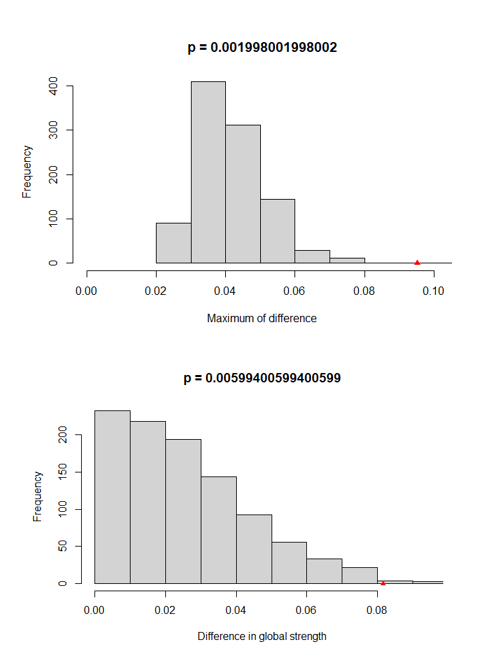


**eFigure 6. Comparison of network attributes of participants in different gender**

Panel above: Plot of bootstrap vale of the diference in network global strength. with significant diference.

Bottom Panel: Plot of bootstrap value of the maximum difference in any of the edge weights (1000permutations)，differences were statisticallv significant.


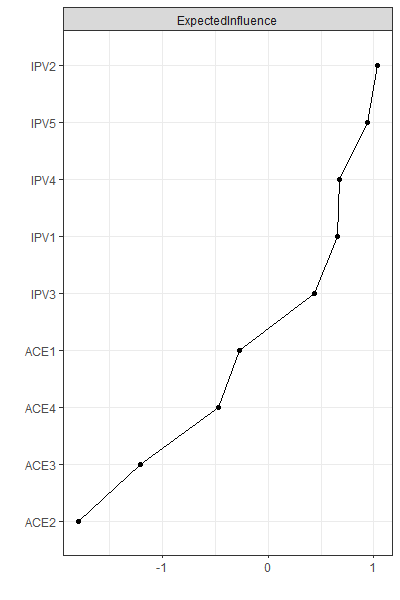


**eFigure 7. EI values of nodes in Male network**


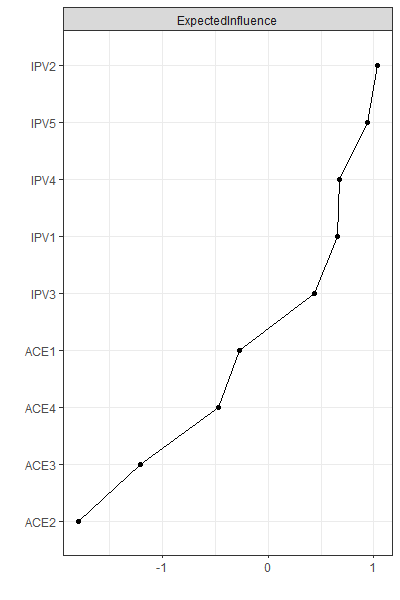


**eFigure 8. EI values of nodes in Female network**


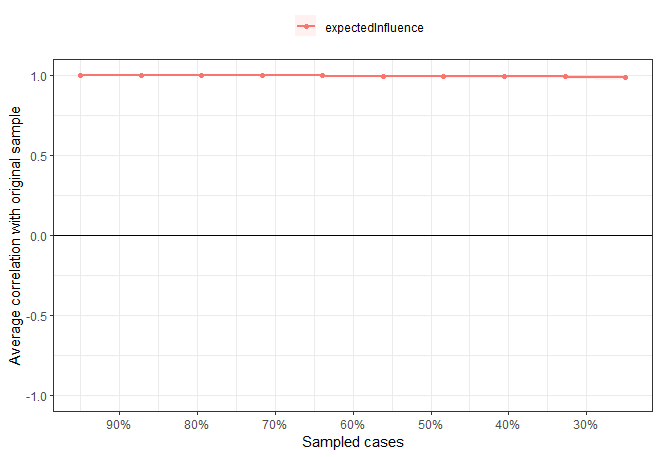


**eFigure 9. Estimating the stability of the network structure using the case-drop subset bootstrap method in Male**


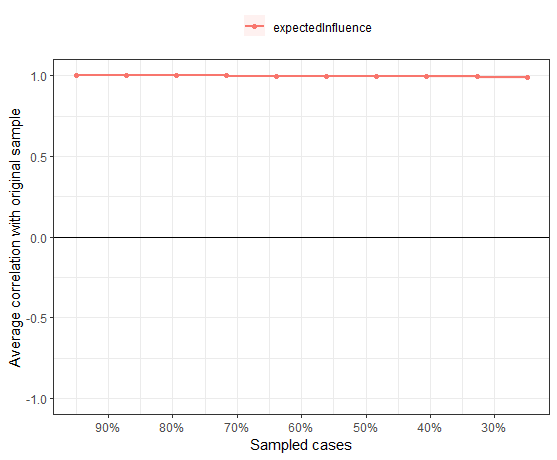


**eFigure 10. Estimating the stability of the network structure using the case-drop subset bootstrap method in Female**


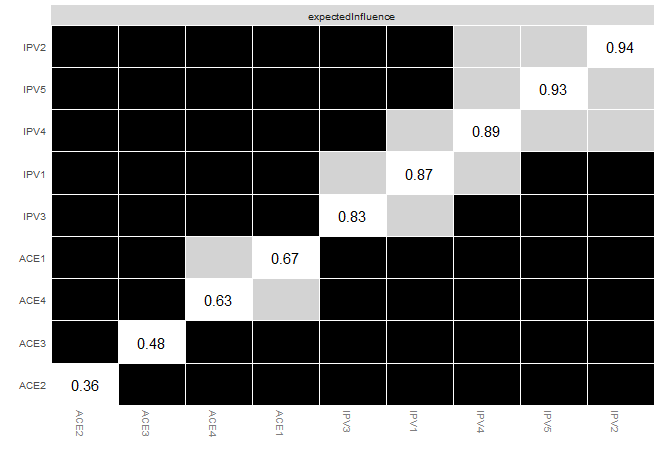


**eFigure 11. Difference-in-difference test for node EI** **in Male**

**
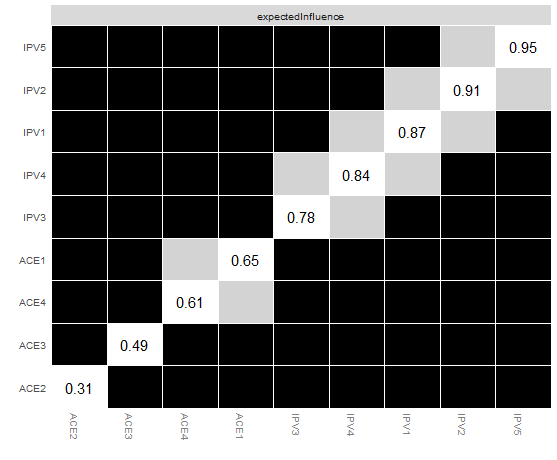
**

**eFigure 12. Difference-in-difference test for node EI in Female**


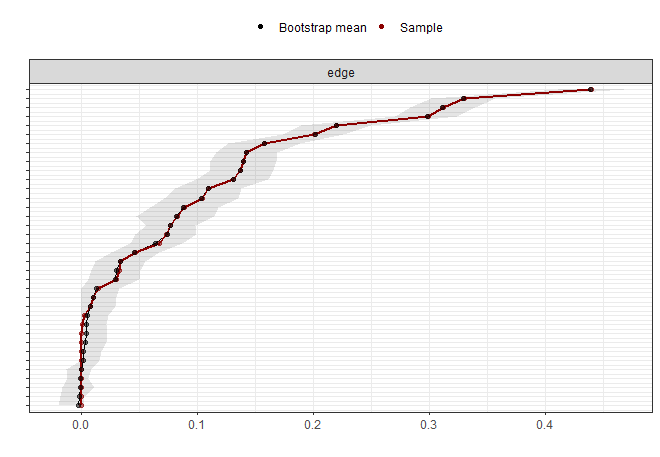


**eFigure 13. Bootstrap Difference Test of Network Edge Weights in Male**

**
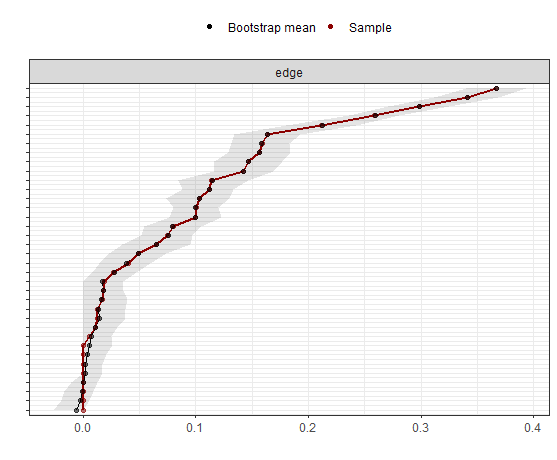
**

**eFigure 14. Bootstrap Difference Test of Network Edge Weights in Female**

**eReferences**

**Wang Y-F, You G-Y, Han T, Liu Y, Li J, Ji X and Xie X-M** (2023) Network analysis of comorbid depression, suicidality and biomarkers on HPA axis among mood disorder patients to psychiatric emergency services. *Translational Psychiatry* **13**(1), 203. https://doi.org/10.1038/s41398-023-02503-5.

**Yuan D, Wu J, Li S, Zhang R, Zhou X and Zhang Y** (2023) Network analysis of cold cognition and depression in middle-aged and elder population: the moderation of grandparenting. *Frontiers in Public Health* **11**, 1204977. https://doi.org/10.3389/fpubh.2023.1204977.
